# Language by mouth and by hand

**DOI:** 10.3389/fpsyg.2015.00078

**Published:** 2015-02-16

**Authors:** Iris Berent, Susan Goldin-Meadow

**Affiliations:** ^1^Phonology and Reading Lab, Department of Psychology, Northeastern UniversityBoston, MA, USA; ^2^Goldin-Meadow Laboratory, Department of Psychology, University of ChicagoChicago, IL, USA

**Keywords:** sign language, universal grammar, modality, language evolution, rules, home signs, emerging sign langauges, lexical access

What is the basis of the human capacity for language: Is language shaped only by sensorimotor constraints and experience, or are some aspects of language universal, abstract, and potentially amodal? The set of papers assembled in this collection represent state of the art research on this age-old set of questions.

To gauge the universality of language structure and its abstraction, the first group of papers examines the grammatical organization of mature languages across modalities. The papers by Baus et al. (2014) and Guellai et al. (2014) suggest that, despite marked differences in modality, the phonology of signed and spoken languages share aspects of design. Specifically, Baus and colleagues demonstrate that syllable-like units are extracted by signers automatically even when the task does not demand it. Using a similar interference paradigm, Guellaï and colleagues show that speakers (of Italian) automatically extract prosodic structure and use manual gestures to help them do it; the cues to prosody that are found in co-speech gesture play a role in disambiguating the syntactic structure of the speech they accompany. The typological survey described in Napoli and Sutton-Spence (2014) extends the study of grammatical universals to syntax. Like spoken languages, sign languages overwhelmingly favor subject-first structures (i.e., SOV and SVO). Unlike spoken languages, however, sign languages show a strong preference for the SOV over the SVO order. This aspect of grammatical organization may thus be influenced by modality, although the fact that signed and spoken languages differ not only with respect to modality but also with respect to age (i.e., spoken languages are older than sign languages) makes it difficult to pinpoint the source of this difference.

Further insights into grammar and its origins are presented in papers on the genesis of sign languages in Deaf communities and in individual homesigners (deaf individuals who have not been exposed to an established sign language and who use their own homemade gestures to communicate with the hearing individuals in their worlds). Given the poverty of linguistic input available to these individuals, and the fact that the manual modality affords iconic depiction, we might expect emerging sign languages to be overwhelmingly iconic. But the role of iconicity is actually far more constrained and nuanced than one might have presumed.

Considering homesigns, Coppola and Brentari (2014) find that the spontaneous emergence of morphophonology in an individual homesigning child mirrors the organization of mature sign languages (i.e., greater finger complexity in Object-handshapes than in Handling-handshapes). But remarkably, this abstract grammatical organization emerges *prior* to the arguably more iconic organization of morphosyntax (i.e., associating Object-handshapes with no-agent events and Handling-handshape with agent events). Moving to another example, this time a sign language that is growing up in Nicaragua, Kocab et al. (2015) find that, contrary to naïve expectations, signers do not immediately rely on iconic spatial devices to mark referential shifts, but rely instead on abstract lexical markers. Further glimpses into the spontaneous emergence of abstract syntactic organization can be found in Kastner et al. (2014), who document how prosody is used to mark the kernels of syntactic embedding in Kafr Qasem Sign Language, a sign language emerging in Israel.

The possibility that signed and spoken languages might both rely on abstract grammatical organization brings the ongoing debate between algebraic (symbolic, rule-based) vs. associationist accounts of spoken language into the domain of sign language—what computational mechanisms are used by signers to support linguistic productivity? The papers by Caselli and Cohen-Goldberg (2014), on one hand, and Berent et al. (2014), on the other hand, suggest that a full account of sign language computation (like spoken language computation) requires both systems, hence, “words and rules (Pinker, 1999).” Considering first the evidence for associations, Caselli and Cohen-Goldberg trace lexical competition in sign language to the same set of dynamic associative principles proposed for spoken languages. Nonetheless, Berent et al. find that signers can extend certain phonological generalizations across the board in a rule-governed way—even to novel signs with features that are unattested in their language. Building on past computational work, Berent et al. suggest that generalizations of this sort are the hallmark of powerful algebraic rules that support the capacity for discrete infinity in the manual modality.

Our review has so far highlighted commonalities across different language modalities and different levels of experience. But the effects of modality and experience are undeniable and significant—the papers by Supalla et al. (2014) and Emmorey et al. (2014) underscore some of these effects. Considering first experience, Supalla and colleagues find that language experience shapes language fluency, which, in turn, shapes the quality of signers' working-memory storage—fluent signers retain global semantic structure, less fluent signers focus on lexical detail and linear order. Considering language modality, Emmorey and colleagues find that, even though signed and spoken languages share neural substrates, sign language comprehension and production engages a unique network of sensorimotor regions that are directly linked to the visual/manual channel; sign comprehension uniquely suppresses visual occipital activity, whereas sign production engages parietal regions involved in manual motor simulation.

The final four papers in this volume consider the development of sign languages and their evolution. Morgan (2014) argues that, across modalities, combinatorial structure emerges gradually out of a system that is initially holistic. Lillo-Martin et al. (2014) investigate the developmental of linguistic communication in bimodal bilingual children. Although these children are clearly sensitive to the language of their interlocutors and they modulate their language choice accordingly, the findings nonetheless reveal an overwhelming preference for speech over sign. In contrast, when adult speakers are engaged in a communication game, Fay et al. (2014) find a strong advantage for gestures over speech (alone, or even in combination with gesture)—a finding that the authors attribute to the affordance of the manual modality for iconicity. The gesture advantage in adult speakers does not speak directly to language evolution in humans, but the results are in line with the possibility that proto-language was gestural. How could such a gestural system give rise to the evolution of spoken language? This question is addressed by Woll (2014), who suggests that echo-phonology might provide the missing link.

## Conflict of interest statement

The authors declare that the research was conducted in the absence of any commercial or financial relationships that could be construed as a potential conflict of interest.
